# Placental Malaria Is Rare Among Zanzibari Pregnant Women Who Did Not Receive Intermittent Preventive Treatment in Pregnancy

**DOI:** 10.4269/ajtmh.13-0586

**Published:** 2014-08-06

**Authors:** Marya Plotkin, Khadija Said, Mwinyi I. Msellem, Rachel P. Chase, Natalie Hendler, Asma Ramadhan Khamis, Elaine Roman, Chonge Kitojo, Alanna C. Schwartz, Julie Gutman, Peter D. McElroy

**Affiliations:** Jhpiego Tanzania, Dar es Salaam, Tanzania; Zanzibar Malaria Elimination Programme Zanzibar Ministry of Health, Tanzania; Jhpiego Baltimore, Baltimore, Maryland; Department of International Health, John Hopkins University Bloomberg School of Public Health, Baltimore, Maryland; Department of Medicine, University of California, San Francisco, California; US President's Malaria Initiative and Centers for Disease Control and Prevention Tanzania, Dar es Salaam, Tanzania; Centers for Disease Control and Prevention, Atlanta, Georgia

## Abstract

Zanzibar has transitioned from malaria control to the pre-elimination phase, and the continued need for intermittent preventive treatment during pregnancy (IPTp) has been questioned. We conducted a prospective observational study to estimate placental malaria positivity rate among women who did not receive IPTp with sulfadoxine-pyrimethamine. A convenience sample of pregnant women was enrolled from six clinics on the day of delivery from August of 2011 to September of 2012. Dried placental blood spot specimens were analyzed by polymerase chain reaction (PCR); 9 of 1,349 specimens (0.7%; precision estimate = 0.2–1.1%) were PCR-positive for *Plasmodium falciparum*. Placental infection was detected on both Pemba (*N* = 3) and Unguja (*N* = 6). Placental malaria positivity in Zanzibar was low, even in the absence of IPTp. It may be reasonable for the Ministry of Health to consider discontinuing IPTp, intensifying surveillance efforts, and promoting insecticide-treated nets and effective case management of malaria in pregnancy.

## Introduction

The devastating consequences of malaria in pregnancy (MIP) on maternal and newborn health are well-documented. Pregnant women in areas of both stable and unstable malaria transmission are at increased risk for peripheral and placental *Plasmodium falciparum* infection, with symptomatic infections being more common in areas of unstable transmission.^1^–^4^ The adverse maternal outcomes of MIP in both transmission settings include severe anemia and mortality, with higher case fatality rates in lower-transmission settings.^3^,^5^,^6^ In addition, MIP is associated with low birth weight (LBW; < 2,500 g),^7^ an important risk factors for infant mortality.^2^,^8^,^9^

In areas of stable malaria transmission, the World Health Organization (WHO) promotes a three-pronged strategy to prevent adverse consequences of MIP, including (1) intermittent preventive treatment during pregnancy (IPTp) using sulfadoxine-pyrimethamine (SP), (2) use of insecticide-treated bed nets (ITNs), and (3) effective case management through prompt detection and treatment of malaria and anemia in pregnancy.^10^–^12^ Zanzibar adopted this three-pronged approach in 2004 when malaria transmission was high and stable.^13^ Since 2006, Zanzibar has scaled up long-lasting insecticidal nets (LLINs) and indoor residual spraying (IRS), improved case detection with malaria rapid diagnostic tests (mRDTs), and introduced artemisinin-based combination therapy (ACT). A marked decline in malaria prevalence in Zanzibar over the past decade (from 50% in 2003 to 0.5% in 2012 among children under 5 years of age) has shifted Zanzibar to the malaria pre-elimination phase.^13^,^14^ In response, Zanzibar is now revising its MIP control strategy. One question is whether to continue IPTp. This study aimed to estimate the rate of placental malaria infection at delivery among Zanzibari pregnant women who had not received IPTp to help determine whether IPTp is still warranted in Zanzibar.

## Methods and Materials

### Study area and sites.

Zanzibar is comprised of two main islands approximately 40 km from mainland Tanzania: Unguja (2012 population = 896,721; 1,666 km^2^) and Pemba (2012 population = 406,848; 998 km^2^).^15^,^16^ This study was conducted at a purposive sample of 6 of 38 health facilities in Zanzibar where routine deliveries occur (3 facilities in Unguja [Mnazi Mmoja Hospital, Mwembeladu Hospital, and Kivunge Health Center] and 3 facilities in Pemba [Chake Chake Hospital, Wete Hospital, and Micheweni Health Center]) from August of 2011 to September of 2012. These facilities were engaged in an ongoing program supported by the US Agency for International Development (USAID) designed to improve maternal and newborn health services. Although these six hospitals covered approximately 82% of hospital deliveries on both islands, 50% of women in Zanzibar deliver at home.^17^ The peak rainfall period occurs from March to June, and the peak malaria transmission period is from April to July.^18^

### Eligibility and enrollment.

Residents of Unguja or Pemba delivering at a study facility were considered eligible if their antenatal care (ANC) card indicated they had received no doses of IPTp SP. Women were enrolled 24 hours/day every day of the week by the clinic staff. Consecutive sampling was used to enroll all eligible women during the 12-month study period. However, because of the added burden on the clinic staff, no listing was maintained of women who were eligible but not enrolled. The 12-month enrollment ensured that both low- and high-transmission seasons were represented.

### Procedures.

Women who delivered at the six facilities were screened by examining their ANC card, which contains a designated space for recording doses of IPTp SP received. No additional questions were asked, because eligible clients were typically in active labor. Eligible women who agreed to participate were read an informed consent statement by trained maternity nurses or nurse midwives, and their verbal consent was obtained and recorded. No additional assent options were offered to minors. A brief form, including age of mother, gravidity, hemoglobin or hematocrit and week of gestation at which the measurement was taken, whether it was a singleton or multiple birth, outcome of the birth, and birth weight of the baby, was filled out by the health worker with information extracted from each consenting mother's ANC card. The form was placed into the client's file and transferred to the delivery room with the client.

### Collection of placental samples.

After management of the birth, a trained maternity nurse or nurse midwife at each facility obtained a placental dried blood spot (DBS) specimen from the maternal side of the placenta. A single deep incision was made, and five drops of blood were drawn up with a pipette and dotted onto Whatman 903 Protein Saver Card filter paper labeled only with the woman's study identification (ID) number.^19^,^20^ DBS specimens were labeled, packed with desiccant, and transferred to the University of California, San Francisco for polymerase chain reaction (PCR) to detect infection with *Plasmodium* (all species). Although exact timing was not monitored, collection of each specimen generally occurred within 30 minutes of delivery. After collection of the specimen, the placenta was disposed of per the facility's routine procedures. Multiple births were noted on the client's study form. In the case of twins with one placenta, one ID was issued. In the case of twins with two placentas, each placenta was assigned a unique ID number and tested. Corresponding placental ID numbers were noted on the client form.

### Laboratory analysis.

DNA from each DBS was extracted using the QIAamp DNA Microkit following the manufacturer's instructions (Qiagen, Germantown, MD). *Plasmodium* DNA was amplified by 18S ribosomal DNA PCR, with the species-specific nested round confirming falciparum species.^21^ The presence of PCR product was evaluated using agarose gel electrophoresis stained with ethidium bromide. Testing of control samples extracted from DBSs with known parasite densities confirmed a limit of detection of < 10 parasite/μL.

### Sample size.

The required sample size was calculated to be 1,825 assuming a parasitemia prevalence of 5% with a 95% confidence interval with precision of ±1% point. The sample size was calculated assuming independence of observations, because intracluster correlations could not be assessed, but the expected prevalence was liberally estimated to help account for unknown variables. Initial results indicated prevalence well under the liberal prevalence estimate used in the sample size assumptions, indicating that fewer samples were needed to attain the necessary power for the study, but data collection continued to allow for coverage throughout both the rainy and dry seasons.

### Data processing and analysis.

Client forms were entered into an Access database and date stamped, and hard copies were filed chronologically along with the consent forms. Data on PCR status were provided in an Excel spreadsheet, and the ID numbers were matched between the two files. Cleaned data were exported into SPSS, version 20 and Stata, version 11 for analysis. Although we attempted to enroll all eligible women, given the total number of deliveries and the expected proportion of women who did not receive IPTp (25%),^14^ it is likely that some of the eligible women were not enrolled. Given that the participants in this study were a non-probability sample of all eligible women, the 95% confidence interval given for the proportion of women with placental parasitemia who did not receive IPTp is considered a precision interval rather than a confidence interval.

### Ethics/institutional review.

Ethical approval was provided by the Zanzibar Research Council, Johns Hopkins School of Public Health Institutional Review Board, and the US Centers for Disease Control and Prevention, Atlanta.

## Results

### Patient characteristics.

**Figure 1.**
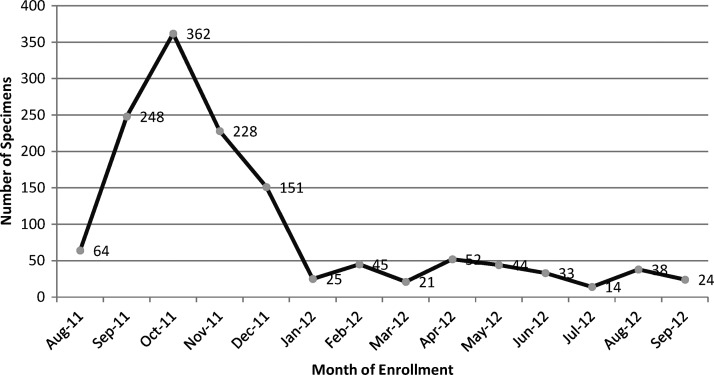
Placental specimens collected by month in Pemba and Unguja, Zanzibar.

In total, 1,411 pregnant women were enrolled at the time of delivery. Data and specimens from 74 enrollees were not analyzed because of mislabeling, duplicate IDs, missing client cards, or loss. After exclusion of these 74 women, our analysis included 1,339 women from Pemba (*N* = 694) and Unguja (*N* = 645). The average age of enrolled women was 27 years (range = 15–48 years), and 433 (32%) women were primigravidae (Table 1). The proportion of primigravidae varied by facility (from 14% of the enrolled women at Mwembeladu to 42% of the enrolled women at Mnazi Mmoja). The enrollees included both singleton (*N* = 1,326) and twin (*N* = 13) pregnancies. Birth outcomes experienced by these women included 1,308 (97%) live births, 22 (2%) stillbirths, and 73 (5%) LBW deliveries (Table 1). The majority of enrolments (67%) occurred in the first 4 months of the study (Figure 1), likely because of SP stockouts preceding and during this period. Overall, 6% of all deliveries at the six facilities were enrolled (ranging from 4% to 15%). Although only 23% of all deliveries at Zanzibar health facilities occurred on Pemba in 2011, deliveries from Pemba comprised 52% of the total enrolment in this study.

### Characteristics of placental parasitemia cases.

In total, 1,349 placental specimens were processed and analyzed for presence of malaria infection by PCR (Table 2). Nine (0.7%; precision estimate = 0.2–1.1%) were positive for *P. falciparum*, with no other *Plasmodium* species detected.

**Figure 2.**
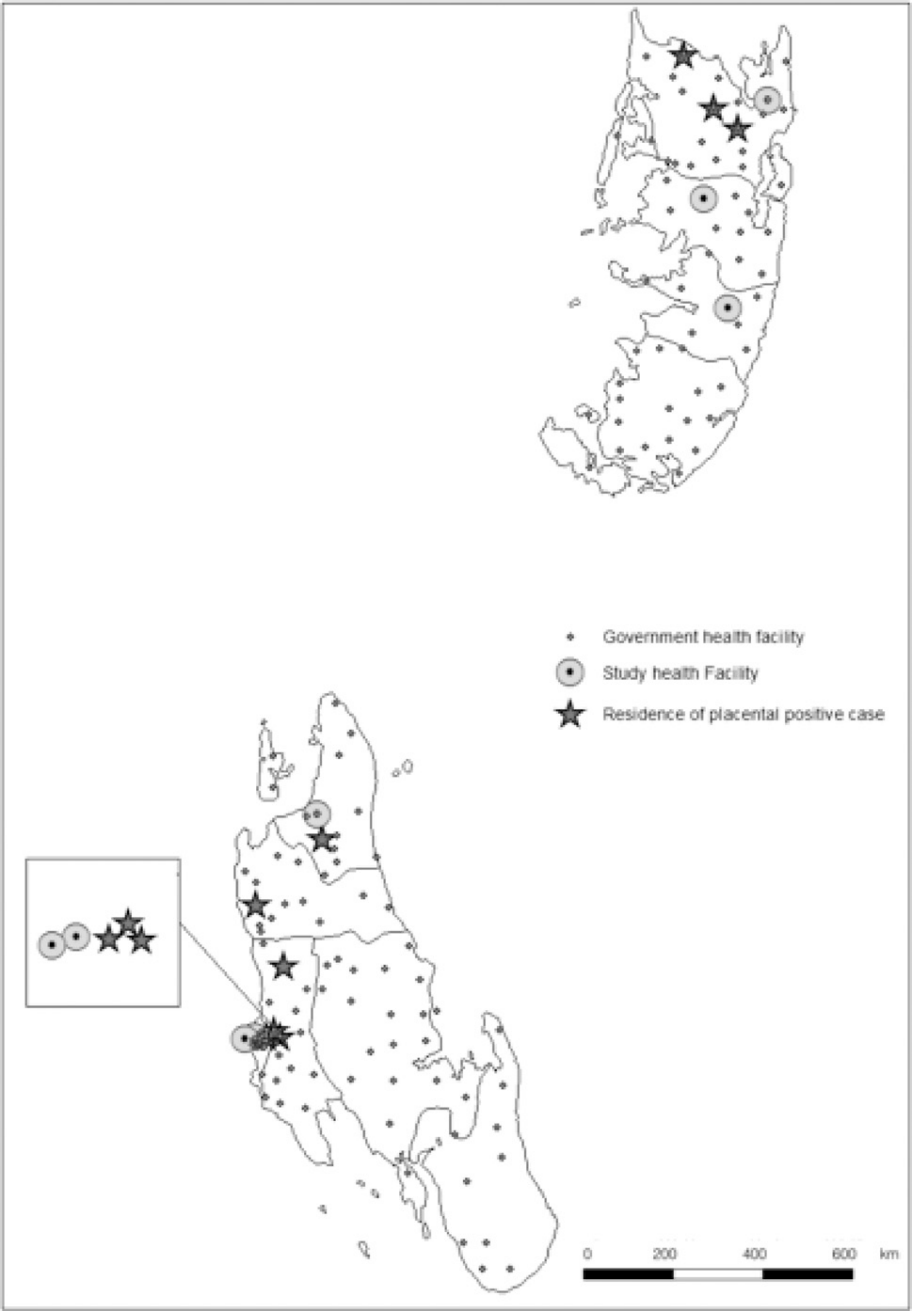
Placental malaria infection cases in Pemba and Unguja, Zanzibar.

Placental infection was detected during 6 of 12 months of enrolment, with no seasonal variation. Unguja and Pemba accounted for six (66%) and three (33%) of the positive placental specimens, respectively (Figure 2). Detection of cases in the low-transmission season (September to December) was likely the result of increased study enrolment eligibility during these months as a result of SP stockouts (Figure 1). Seven (78%) positives originated from two of six enrolment facilities: Mwembeladu Hospital and Micheweni Health Center on Unguja and Pemba, respectively. In Pemba, placental infection (*N* = 3) was only detected at Micheweni, but at least one placental infection was detected at each of three Unguja facilities.

Birth outcomes among nine placental infections included one stillborn infant of LBW (< 2.5 kg). None of the remaining women with placental parasitemia delivered an LBW infant. Three women with placental parasitemia had hemoglobin tests; two women were anemic (hemoglobin < 11 g/dL) (Table 3). The proportion of anemic women at time of delivery was similar in women with and without placental malaria. Three positive specimens came from primigravidae women, proportionate to the percent of primigravidae sampled in the study.

## Discussion

The prevalence of placental malaria at the time of delivery in Zanzibar was very low among women in this study who did not receive IPTp SP, with an infection rate of 0.7% (precision estimate = 0.2–1.1%) by PCR. Data from screening women for peripheral parasitemia at first ANC visit with mRDTs at government health facilities shows a similar low prevalence of 0.2% among 19,724 women in 2011 and 27,186 women in 2012,^22^,^23^ confirming the low risk of MIP in Zanzibar. These data triangulate well with Zanzibar's Malaria Early Epidemic Detection System (MEEDS), which collects weekly data from all government facility outpatient departments. MEEDS reported a malaria positivity rate of 1.2% and 0.9% among all febrile or otherwise symptomatic outpatients tested during 2011 and 2012, respectively (Zanzibar Malaria Control Program, unpublished data).

The low prevalence of malaria overall and particularly, the low prevalence documented in pregnant women who deliver in health facilities raises multiple questions about the use of continuing IPTp SP as part of ANC services in Zanzibar. In areas of moderate to high malaria transmission, the WHO recommends IPTp in addition to ITNs and effective case management. In African countries that have substantially reduced malaria transmission after successful scale up of multiple control efforts, the WHO continues to recommend provision of IPTp.^12^,^24^ Despite this recommendation, Rwanda discontinued their national IPTp program in 2008 because of increased prevalence of SP resistance combined with declining malaria prevalence.^25^ The WHO's Malaria Policy Advisory Committee recently considered an Evidence Review Group (ERG) recommendation to discontinue IPTp SP when malaria transmission has been very low (falciparum malaria population prevalence in children under 15 years of age below 5%) for at least 3 years. The ERG's recommendation was based on findings from two unpublished analyses that suggested that, as transmission falls to very low levels, the fraction of malaria-attributable LBW prevented by IPTp SP continues to decline.^26^ The ERG emphasized that discontinuation of IPTp would require simultaneous maintenance and strengthening of vector control, prompt diagnosis and effective treatment, and surveillance systems to monitor malaria transmission. As of late 2013, the WHO had not changed its recommendation against discontinuation of IPTp, because the threshold value where IPTp SP confers no benefit remains undefined.

The potential for malaria resurgence in Zanzibar remains, given historical observations, population movement, and proximity to mainland Tanzania.^27^ It is reassuring that, since 2007, the population prevalence of malaria in cross-sectional household surveys in Zanzibar has remained below 1% and that, since 2008, MEEDS data indicate positivity rates well below 5% among febrile outpatients. Tanzania mainland has also reduced malaria prevalence among children 6–59 months of age (from 18% in 2007 to 10% in 2012).^14^ However, transmission in the nearby coastal regions Pwani and Tanga (10% and 6% prevalence, respectively, in 2012) remains substantially higher than in Zanzibar. Zanzibar's proximity to the mainland must be taken into consideration in any discussion to discontinue IPTp, particularly considering complexities and costs associated with restarting the IPTp SP program after discontinuation in case that is warranted.

The seasonal increases in malaria transmission and focal hot spots in Zanzibar must also be considered. After the rainy season each year, confirmed cases of malaria rise during May to July. Weekly malaria positivity rate has exceeded 5% (threshold for pre-elimination) one time per year in both 2010 and 2012 (maximum weekly positivity of 10% among patients ≥ 5 years of age).^28^ Thus, although the overall annual malaria prevalence and outpatient positivity rates remain extraordinarily low, periodic localized increases in malaria transmission do occur. As a strategy for prevention of MIP, however, it is unlikely that administering SP to all Zanzibari pregnant women throughout the year would significantly modulate risk during these brief periods of increased malaria transmission.

Because of Zanzibar's proximity to the mainland, approaches to control of MIP must include a strong component of surveillance and maintenance of high ITN use as well as ensure access to prompt diagnosis and effective treatment. The MEEDS surveillance system does not adequately monitor MIP for two reasons: (1) the entry point to MEEDS is limited to outpatient departments (OPDs), whereas symptomatic ANC clients may report to ANC clinics, and (2) the system does not capture pregnancy status of adults who present to OPDs. Enhanced surveillance of MIP could be achieved through inclusion of ANC malaria test results into MEEDS and modification of MEEDS to allow reporting of outpatient test results stratified by pregnancy status.

Among the alternatives to IPTp SP are two screening and treatment approaches: (1) intermittent screening and treatment of MIP (ISTp), in which every woman is tested at every ANC visit, and (2) single screen and treatment (SSTp), in which all pregnant women are tested at the first ANC visit and only symptomatic women are screened thereafter. Data comparing ISTp with IPTp from a single trial in Ghana show similar reductions in maternal morbidity, peripheral parasitemia, and LBW with ISTp (using SP or amodiaquine-artesunate) compared with IPTp SP.^29^ Additional studies comparing the effectiveness of IPTp SP with ISTp with artemether-lumefantrine are underway in East Africa, and results are expected in 2015 (ClinicalTrials.gov Identifier NCT01084213). SSTp is currently the national policy in Indonesia, where an ongoing study is comparing SSTp and ISTp using dihydroartemisinin-piperaquine for treatment. However, these data are not available to address the current decision-making process for a revised MIP strategy in Zanzibar. Another complication is that neither ISTp nor SSTp is currently endorsed by the WHO.

Both IPTp and widespread screening in ANC have cost implications, including commodity, logistics, training, supervision, and monitoring costs. Screen and treat strategies are likely to be more costly than IPTp SP given the higher cost of the RDTs compared with SP. In a setting with virtually no cases of malaria, these costs must be weighed against the potential risks and benefits to mothers and newborns, including the potential for serious adverse drug reactions related to SP use (estimated at 11.6–25.0 events per 100,000 exposures with a mean cost per episode of US$24.15 [range = US$0–226.04]).^30^ Given the cost implications of screen and treat programs, a setting such as Zanzibar, with a prevalence persistently below 1%, may reasonably consider discontinuing IPTp SP without implementing a screen and treat program. Such a move would necessitate maintaining and improving the other pillars of malaria prevention, including use of ITNs, effective case management, and additional enhancement of the existing surveillance system.

ITNs remain a critical part of the overall malaria control strategy supported by the Roll Back Malaria Partnership. ITNs not only provide protection to pregnant women but also reduce all-cause mortality among infants and young children by up to 20%.^31^ High ITN use at the community level has also achieved malaria transmission reductions.^32^ Provision of ITNs to pregnant women is one avenue to maintain continuous high coverage. Furthermore, in a large randomized controlled trial in a low-transmission setting in Uganda, ITNs and IPTp provided equivalent benefit to pregnant women.^33^ This finding suggests that, where ITN distribution forms part of the basis for general malaria control, it may be reasonable to discontinue IPTp and continue high ITN coverage as one strategy to avert MIP.

### Limitations.

This study is limited to the cross-sectional prevalence of placental parasitemia among a consecutive sample of women delivering in health facilities in Zanzibar, but more than one-half of the women in Zanzibar deliver at home. The representativeness of our study sample may be questioned, because it is not unreasonable to presume that women delivering at home are less likely to receive IPTp and thus, more likely to be at risk for malaria infection. However, the high level of concordance among the results of our study, MEEDS, and findings from ANC screening provides reassurance that these results are valid. Receipt or non-receipt of IPTp SP was determined solely by reviewing the client ANC card at delivery, which means that the quality of information for enrolment eligibility was limited by the recording practices of ANC providers. In most cases where women had not received IPTp SP, it was indicated rather than just an absence of a marking on the card. Because SP is not widely available outside of ANC (in 2011, the Zanzibar Food and Drug Board banned the sale of SP and artemisinin monotherapies from private drug shops), the proportion of women who could have received SP outside of ANC (and thus, been misclassified as not receiving SP) is negligible. National survey data confirm that the proportion of women reporting receipt of any antimalarial in pregnancy (85%) is virtually identical to the proportion reporting having received SP from the ANC (84%).^14^ Because of SP stockouts, most women were enrolled in low-transmission months (September to December), potentially resulting in lower prevalence of placental malaria. Although we attempted consecutive enrollment of all eligible women, it is likely that it did not occur. Because no record was maintained of women who were not enrolled, to minimize the study-associated workload among busy labor/delivery ward staff, the exact number of eligible women remains uncertain. However, failure to enroll eligible women was likely related to the workload at the time that the woman presented, which is unlikely to be related to the outcome of interest. This limitation did not likely compromise the generalizability of our results to all pregnant women who did not receive IPTp delivering at these facilities. Because the facilities were selected purposively from the facilities supported by a USAID-funded program designed to improve maternal and newborn health services, the generalizability of the findings to all pregnant women who deliver at health facilities in Zanzibar cannot be assured. The use of histopathology, rather than PCR alone, would have allowed us a better estimation of the prevalence of malaria throughout a longer proportion of the pregnancy; however, it was beyond the scope of this study. Finally, because this study did not enroll women who did receive IPTp SP, no information was collected on the current protective efficacy of IPTp SP in Zanzibar.

### Conclusion.

As malaria transmission in Zanzibar reaches the pre-elimination phase, MIP policy and strategies must be suitably adjusted. This study found a negligible level of malaria among women who had not received IPTp SP. Given the extremely low levels of malaria in both the general population and pregnant women, discontinuing IPTp SP is one reasonable option for Zanzibar to consider. A shift away from IPTp SP as part of Zanzibar's MIP control strategy should only be considered if increased commitment is made to ensure uninterrupted, high-quality access to malaria diagnosis and treatment during pregnancy combined with enhanced measures to drive current transmission levels down closer to zero. We further recommend enhanced surveillance of MIP through reinforcement of the existing surveillance system to ensure that cases among pregnant women are reported from both ANC and outpatient settings in a timely manner. Efforts should continue to ensure high ownership and use of ITNs, particularly among women of reproductive age. Findings from ongoing studies in higher-transmission areas on the cost effectiveness of integrating screen and treat programs into ANC services may not be directly relevant in Zanzibar, where prevalence of malaria is much lower. The Zanzibar Ministry of Health should consider an internal review of costs and findings from surveillance to inform on whether the cost of screening every pregnant woman in ANC is a sound public health investment.

## Figures and Tables

**Table 1:** Characteristics of pregnant women enrolled at delivery (*N* = 1,339) on Pemba and Unguja islands of Zanzibar in 2011–2012

Island/health facility	Annual deliveries (2011)*	Enrolled women with specimen analyzed, *n*	Mean age (range), years	Primigravid, *n* (%)	Twin pregnancies, *n* (%)
Unguja					
Mnazi Mmoja	10,338	375	27 (15–46)	160 (42)	5 (1)
Mwembeladu	5,665	205	28 (17–46)	29 (14)	2 (1)
Kivunge	1,420	65	25 (16–42)	25 (37)	2 (3)
Pemba					
Chake Chake	2,838	415	27 (15–48)	132 (32)	3 (1)
Wete	1,607	189	27 (16–48)	50 (26)	1 (1)
Micheweni	645	90	25 (17–42)	37 (41)	0 (0)
Total	22,563	1,339	27 (15–48)	433 (32)	13 (1)

* These figures represent deliveries during the 2011 calendar year.

**Table 2:** Distribution of malaria-positive placental specimens (*N* = 9) among study participants in 2011–2012

Characteristic	Positive, *n* (%)	Total, *n*	*P* value
Season			0.63
September to December	6 (1)	1,014	
January to April	2 (1)	143	
May to August	1 (1)	192	
Facility			0.004
Unguja			
Mnazi Mmoja	1 (< 1)	380	
Mwembeladu	4 (2)	207	
Kivunge	1 (1)	67	
Pemba			
Chake Chake	0 (0)	416	
Wete	0 (0)	189	
Micheweni	3 (3)	90	
Gravidity			1.0
Primigravidae	3 (1)	433	
Multigravidae	6 (1)	916	

**Table 3:** Characteristics of malaria-positive placental specimens (*N* = 9) among study participants in 2011–2012

Case number	Island	Health facility	Month of delivery	Parity	Birth weight (kg)
1	Pemba	Micheweni	September 2011	1	2.5
2	Unguja	Mwembeladu	October 2011	6	3.8
3	Unguja	Mwembeladu	October 2011	3	2.8
4	Unguja	Mnazi Mmoja	November 2011	5	4.2
5	Unguja	Mwembeladu	December 2011	2	3.1
6	Unguja	Kivunge	December 2011	1	3.5
7	Pemba	Micheweni	April 2012	1	2.5
8*	Pemba	Micheweni	April 2012	3	1.8
9	Unguja	Mwembeladu	May 2012	4	2.5

* Macerated stillbirth.
